# Luminescent Sensor Based on Ln(III) Ternary Complexes for NAD(P)H Detection

**DOI:** 10.3390/molecules25184164

**Published:** 2020-09-11

**Authors:** Filip Smrčka, Přemysl Lubal

**Affiliations:** Department of Chemistry, Faculty of Science, Masaryk University, Kamenice 5, 625 00 Brno, Czech Republic; 394208@mail.muni.cz

**Keywords:** Ln(III) complexes, macrocyclic ligands, Ln(III) luminescence in VIS/NIR range, antenna ligand, NAD(P)H determination, enzyme probe

## Abstract

Ln(III) complexes of macrocyclic ligands are used in medicinal chemistry, for example as contrast agents in MRI or radiopharmaceutical compounds, and in diagnostics using fluorescence imaging. This paper is devoted to a spectroscopic study of Ln(III) ternary complexes consisting of macrocyclic heptadentate DO3A and bidentate 3-isoquinolinate (IQCA) ligands. IQCA serves as an efficient antenna ligand, leading to a higher quantum yield and Stokes shift (250–350 nm for Eu, Tb, Sm, Dy in VIS region, 550–650 nm for Yb, Nd in NIR region). The shielding-quenching effect of NAD(P)H on the luminescence of the Ln(III) ternary complexes was investigated in detail and this phenomenon was utilized for the analytical determination of this compound. This general approach was verified through an enzymatic reaction during which the course of ethanol transformation catalyzed by alcohol-dehydrogenase (ADH) was followed by luminescence spectroscopy. This method can be utilized for selective and sensitive determination of ethanol concentration and/or ADH enzyme activity. This new analytical method can also be used for other enzyme systems coupled with NAD(P)H/NAD(P)^+^ redox pairs.

## 1. Introduction

**Figure 1:**
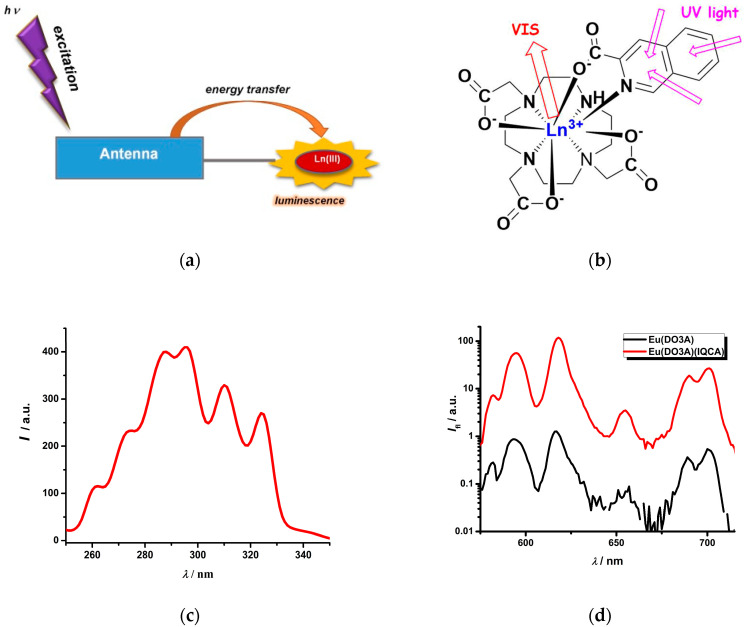
The antenna effect of ternary Ln(III) complexes with DO3A macrocyclic and 3-isoquinolinecarboxylate (IQCA) ligands (**a**,**b**). The excitation spectra (**c**) of Eu(DO3A)(IQCA) (*λ*_em_ = 618 nm) and emission spectra (**d**) of both binary and ternary complexes (*λ*_exc_ = 325 nm). The concentration of the Eu(III) complex is about 0.1 mM.

The specific spectroscopic, electrochemical and magnetic properties of Ln(III) ions make them perfect candidates for use in many chemical, biological and environmental systems. Ln(III) ions in their complexes exhibit unique luminescence properties because of sharp and characteristic emission bands whose positions are not influenced by the physico-chemical properties of the ligand [1,2,3,4,5]. In addition, the luminescence spectra of Ln(III) complexes can be recorded in time-resolved mode due to relatively long lifetimes (in the μs-ms scale). This enables the filtering of their signal from the in vivo background originating from organic molecules with luminescent decay on the ns time scale [1,2,3,4,5,6,7,8]. The excited states of Ln(III) ions in their complexes are not quenched by O_2_ molecules but are influenced by water molecules coordinated to the Ln(III) ion [1,2,3,4,5,6,7,8]. Therefore, their luminescence intensity is higher in complexes than in aqua ions. The introduction of a chromophoric group into the ligand significantly increases the luminescence of Ln(III) complexes as consequence of the so-called antenna effect, through which energy strongly absorbed by the chromophoric ligand is efficiently transferred to the Ln(III) ion. This is followed by irradiation of luminescence, with spectra that are characteristic for each Ln(III) ion (see Figure 1a) [1,2,3,4,5,6,7,8,9,10]. This phenomenon can be utilized for the structural design of Ln(III) complexes with applications in biology and medicine—clinical diagnostics [7,8,9,10,11,12,13], mostly as luminescent sensors and probes [12,14,15,16].

The Ln(III) complexes employed in vivo applications should have high thermodynamic stability as well as kinetic inertness to ensure that toxic Ln(III) ions are not released from their complexes; these ions are capable of substituting Ca(II) ion(s) bound in biomolecules [5]. The most suitable ligands for the tight binding of Ln(III) ions are highly rigid macrocyclic compounds which fulfil the above-mentioned properties due to the macrocyclic effect [17,18,19]. When the coordination number of the Ln(III) ion (usually about 8–9) is equal to denticity of macrocyclic ligand, complexes with 1:1 stoichiometry are formed [5,14,15,16,17,18,19,20,21,22,23,24]. For these reasons, Ln(III) complexes with macrocyclic ligands (mainly DOTA derivatives) are commonly utilized in medicinal chemistry as radiopharmaceuticals (^90^Y, ^153^Sm, ^166^Ho, ^177^Lu) [18,25] and contrast agents for MRI (Gd) [5,18,26,27]. When the denticity of the macrocyclic ligand is lower than that of the Ln(III) ion, there is a higher tendency to form ternary complexes. This kinetically inert binary Ln(III) complex is capable of binding another ligand bearing a functional group with specific physico-chemical properties (e.g., chromophore, fluorophore, electroactive group, etc.) and thus this Ln(III) ternary complex can be tailored according to the desired applications [10,17,28,29,30]. The highly luminescent ternary [Eu(DO3A)L] and [Tb(DO3A)L] complexes (see Figure 1b, L = picolinic/3-isoquinolic acid) can be given as an example [28,29,30]. In these complexes, the sensitization of Ln(III) luminescence by a fluorophore is achieved via efficient energy transfer from the ligand to the Ln(III) ion. The luminescent ternary Eu(III) complex is also electroactive due to possibility of the reduction pathway Eu(III) → Eu(II) occurring alongside the redox processes taking place on the fluorophoric group [29].

Nicotinamide adenine dinucleotide consists of two nucleotides (adenine, nicotinamide) joined by a common phosphate group. Its phosphorylated analogue in the 2′ position on the ribose ring is now recognized as a universal energy carrier performing reversible two-electron transfers in a variety of essential metabolic reactions [31,32]. Thus, NAD(P)H/NAD(P)^+^ are considered compounds of paramount biological importance because they serve as cofactors in many metabolic pathways. Both are involved in redox reactions because of their ability to carry electrons from one reaction to another (NAD(P)H and NAD(P)^+^ are reducing/oxidizing agents able to donate/accept electrons) and therefore they are used as substrate-coenzymes of redox enzymes in chemical reversible reactions [31,32]. NAD can be converted into the NAD-phosphate coenzyme, which has usually analogous redox chemistry and serves as a cofactor in anabolic metabolism, e.g., reductive synthetic reactions—synthesis of fatty acids and steroids as well as in oxidant production for antioxidant protection [31,32]. On the other hand, the NAD redox pair is generally involved in catabolic processes [31], e.g., Krebs citric acid cycle, glycolysis, β-oxidation of fatty acids, etc. They also participate in the addition/elimination of chemical groups to or from proteins, e.g., in post-translational modifications; therefore, enzymes participating in NAD metabolism are very often considered targets for the development and testing of new drugs [32]. The concentration of NAD species can be estimated to tenths-units of mM scale [32,33,34]. The NAD(P)^+^/NAD(P)H ratio is an important cell parameter as it reflects both metabolic activity and the health of cells. The NAD^+^/NADH ratio (usually > 1) is a complex parameter because of the overall contribution of several key enzymes. Conversely, the NADP^+^/NADPH ratio is much lower than one, meaning that NADPH is the dominating species of this coenzyme. This ratio for the same concentration of both species can also be represented as a conditional standard redox potential (pH = 7) for the following redox reaction:

NAD(P)^+^ + H^+^ + 2e^−^ ⇔ NAD(P)H (R1)

whose value does not differ dramatically (−320 mV for NAD^+^, −324 mV for NADP^+^ [32]). Due to different spectral properties of both species, this ratio can be used to monitor enzyme activity by molecular spectroscopy. The direct spectroscopic determination of NAD(P)H is based on the fact that the absorption/excitation band(s) of NAD(P)H are between 280–360 nm, with the maximum occurring at 340 ± 30 nm and the emission band at 460 ± 50 nm. Any absorption/emission band of NAD(P)^+^ could not be detected [34,35].

A typical example with NAD^+^ cofactor is alcohol dehydrogenase (ADH, EC 1.1.1.1), which can catalyze the reversible oxidation of alcohol to the corresponding aldehydes and ketones with the reduction of the nicotine adenine dinucleotide [35,36]. The oxidative reaction of ethanol to acetaldehyde coupled with the reduction of NAD^+^ to NADH is catalyzed by the ADH enzyme:

CH_3_CH_2_OH + NAD^+^ → CH_3_CHO + NADH + H^+^ (R2)

This leads to a change in the NAD^+^/NADH ratio, which helps follow the metabolic effects of ethanol in the human body [36]. In addition, it can be also used for the determination of ethanol in food, alcoholic drinks, etc. Some examples are given elsewhere [35,37,38,39,40,41].

In this paper, we first studied the antenna effect of Ln(III) ternary complexes which emit characteristic luminescence spectra in the visible and NIR wavelength ranges. The NAD(P)H quenching phenomena on Ln(III) complex luminescence were investigated. In the last part, the results related to the quenching effects were verified for possible applications in bioanalysis using enzymes with the NAD(P)H/NAD(P) redox couple.

## 2. Results and Discussion

### 2.1. Ln(III) Complexes with an Antenna Ligand

The macrocyclic ligands H_2_DO2A and H_3_DO3A, which differ in their denticity (hexadentate for DO2A^2−^ and heptadentate for DO3A^3−^), are capable of forming Ln(III) complexes with high thermodynamic stability and kinetic inertness [19,20,21,23,24]. The formation of these Ln(III) complexes with characteristic sharp-peak emission spectra is accompanied by an increase of Ln(III) ion luminescence intensity due to the release of water molecules coordinated to Ln(III) ion [24,28]:

[Ln(H_2_O)_9_]^3+^ + L*^z^*^−^ ↔ [LnL(H_2_O)_2-3_]^3-*z*^ + (6-7) H_2_O (R3)

Two or three water molecules are still bound to the Ln(III) ion; their number depends on the coordination number of the Ln(III) ion as well as on the denticity of ligand. Some of these coordinated water molecules can be released through the formation of ternary complexes with another bidentate ligand [28,29]:

[LnL(H_2_O)_2-3_]^3-*z*^ + Y^−^ ↔ [LnLY]^2-*z*^ + (2-3) H_2_O (R4)

If this ligand (Y) is a strong chromophore, it can transfer absorbed energy from its excited state directly to the Ln(III) ion. This is then followed by an emission that is characteristic for each ion. Thus, the ligand Y can serve as an antenna for the collection of absorbed light followed by energy transfer to the Ln(III) ion (see Figure 1a,b) due to fact that no water molecules (efficient quenching agents) are bound to Ln(III) ion. Therefore, this is a complex process dependent on the physico-chemical properties of both the Ln(III) ion and ligand [1,2,3,4,5,6,7,10,28,29] (see Figure 1b).

We have recently shown [28,29,30] that the structurally similar picolinic (PA) and 3-isoquinolinic (IQCA) acids are sensitive antenna ligands for both Eu(III) and Tb(III) complexes with the DO3A ligand. IQCA is slightly better than PA since the excitation band becomes broader (Figure 1c) and some new bands appear. One can see that the excitation wavelength of the less energetic band for the Eu(III) complex is significantly shifted by 40 nm (from 286 nm to 326 nm) (Figure 1c, [28,29]). Another advantage of IQCA is a lower self-quenching effect [28]. All these factors lead to an increase in the quantum yield of about two orders of magnitude (see Figure 1d, [23,28,29]).

In this paper, we investigated the antenna effect for other Ln(III) ternary complexes usually emitting luminescence in the visible (Sm, Dy) and NIR region (Nd, Yb). In literature, some examples of binary Ln(III) complexes with ligands bearing antenna functional groups and emitting in the NIR region can be found [3,14,42,43]; therefore, photophysical studies for analogous ternary Ln(III) complexes are needed. In addition, the study of Nd(III) and Yb(III) ternary complexes is interesting from a structural point of view since the Nd(III) ion is the largest of the studied Ln(III) ions with a coordination number of 9. On the contrary, the Yb(III) ion is the smallest and therefore, its coordination number should be 8 [1,2,3,4,5].

**Figure 2:**
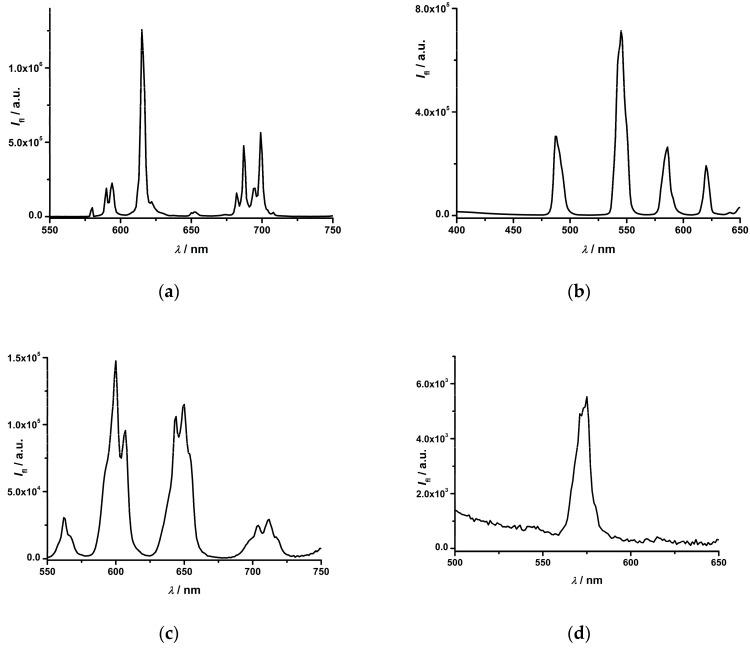
The antenna effect of ternary Eu(III) (**a**), Tb(III) (**b**), Sm(III) (**c**) and Dy(III) (**d**) complexes with DO3A macrocyclic and IQCA ligands (*λ*_exc_ = 325 nm, pH = 7.5 (40 mM HEPES), *c*_Ln_ ~ 0.2 mM, *c*_DO3A_ ~ 0.3 mM, *c*_IQCA_ ~ 0.35 mM).

The Eu(III) and Tb(III) ternary complexes show rich emission spectra where the position of peaks does not depend on the chemical character of the antenna ligand. The most emissive peaks are at 618 nm and 545 nm for Eu(III) and Tb(III) complexes (Figure 2a,b), respectively, and they belong to the ^5^D_0_ → ^7^F_2_ and ^5^D_4_ → ^7^F_5_ transitions [42]. In comparison with [Ln(DO3A)(PA)] complexes [28,29], the more intensive peak in the 650–700 nm region (^5^D_0_ → ^7^F_4_ for Eu) and 575–625 nm region (^5^D_4_ → ^7^F_4_ for Tb) in both emission spectra can be observed as a consequence of the lower flexibility and symmetry of the ternary Ln(III) complexes [5,42]. Furthermore, the peaks of the Eu(III) complex are split, which supports this hypothesis. For the Sm(III) ternary complex, a similarly rich emission spectrum, but with lower intensity (about 13% of the Eu(III) complex), was recorded (Figure 2c). By contrast, a “poorer” emission spectrum with the lowest intensity was measured for the Dy(III) complex (Figure 2d). Generally, the maximum values for Stokes shifts for all complexes are about 375 nm (Eu, Sm), 300 nm (Tb) and 250 nm (Dy).

**Scheme 1:**
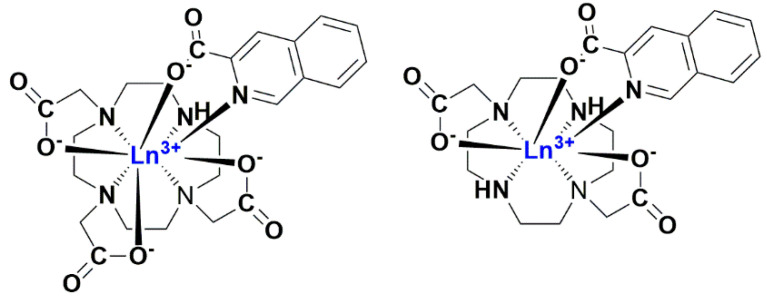
The structural formulas of the discussed Ln(III) ternary complexes (Ln = Nd or Yb).

**Figure 3:**
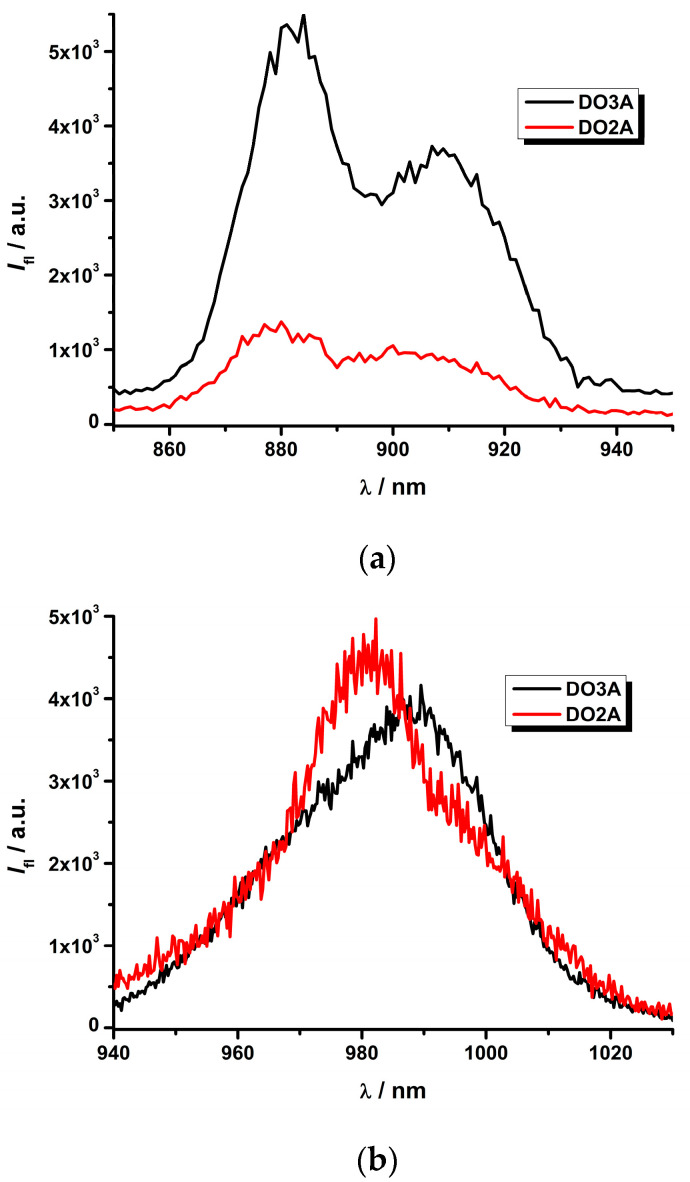
The antenna effect of ternary Nd(III) (**a**) and Yb(III) (**b**) complexes with DO2A and DO3A macrocyclic and IQCA ligands (*λ*_exc_ = 325 nm, pH = 7.5 (40 mM HEPES), *c*_Ln_ ~ 0.2 mM, *c*_DO3A_ ~ 0.3 mM/*c*_DO2A_ ~ 0.4 mM, *c*_IQCA_ ~ 0.35/0.7 mM for Ln(III) complexes with DO3A and DO2A ligands.

The Nd(III) and Yb(III) ternary complexes (see Scheme 1) were prepared in order to investigate the overall coordination number of these Ln(III) ions in their ternary complexes (the emission spectra are displayed in Figure 3). One can observe the most intensive peaks in the emission spectra for the Nd(III) complexes at 880 and 910 nm (^4^F_3/2_ → ^4^J_9/2_ transitions) and the Yb(III) complexes at 980 nm (^2^F_5/2_ → ^2^J_7/2_ transition) [42]. While the highest luminescence intensity was observed for the [Nd(DO3A)(IQCA)]^−^ complex, the opposite phenomena was found for both Yb(III) ternary complexes, where [Yb(DO2A)(IQCA)] and [Yb(DO3A)(IQCA)]^−^ exhibited almost the same luminescence intensity. The explanation is that the Yb(III) ion in its complexes has a total coordination number of 8 while the Nd(III) ion has 9 (see Scheme 1). In both cases, it can be assumed that there is no water molecule coordinated to the Ln(III) ion and therefore no quenching effect is observed. In addition, it is interesting to note that the Stokes shift for both Ln(III) complexes is larger (Yb 655 nm, Nd 555–575 nm) in comparison to previous Ln(III) complexes emitting in visible region (compare 250–375 nm).

The formation of ternary Ln(III) complexes in solution was verified through several experimental techniques. Firstly, high-resolution mass spectra were recorded for Eu(III), Tb(III) and Yb(III) complexes (Figures S1–S3 in Supplementary Materials). The agreement between the predicted and experimental *m*/*z* values for these molecular species was excellent, with values differing by less than 1 ppm (Figures S1–S3). In addition, the *m*/*z* pattern typical for Ln(III) ions with more natural isotopes was found in the mass spectra (Figures S1–S3). Secondly, time-resolved luminescence spectroscopy showed the decrease of the number of water molecules (Δ*q* = 0.8) calculated from luminescence decays for the [Eu(DO3A)] and [Eu(DO3A)(IQCA)] complexes (0.372 ms [23] vs. 0.512 ms—see Figure 6b, leading to the *q* values equal to 2.1 and 1.3). Further, a significant increase in the quantum yield as a consequence of the antenna effect was also observed for these complexes (0.024% [23] vs. 6.9%—estimation using [Eu(DPA)_3_]^3−^ complex as standard). The same antenna effect was observed for the binding of bidentate salicylic acid by the [Tb(DO3A)] complex accompanied by Δ*q* = 0.4 and about 22-times higher *I*/*I*_0_ ratio [44]. The cyclic voltammetry plot of the [Eu(DO3A)(IQCA)] complex also shows the reversible binding of the IQCA ligand [29].

### 2.2. Photophysical Study of Ln(III) Ternary Complexes in the Presence of the NADPH Ligand

**Figure 4:**
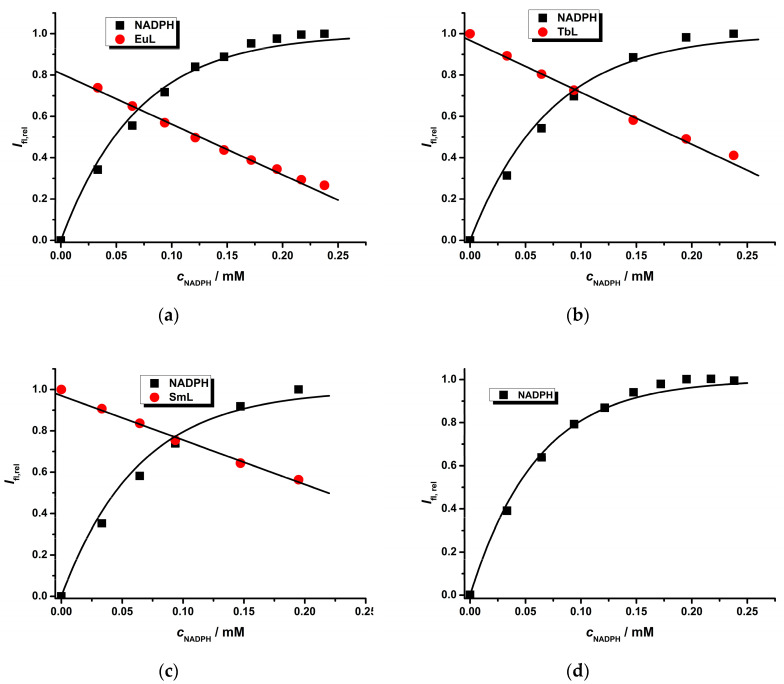
The quenching effect of NADPH on the luminescence intensity of ternary Eu(III) (**a**), Tb(III) (**b**) and Sm(III) (**c**) complexes with DO3A macrocyclic and IQCA ligands (*λ*_exc_ = 325 nm, pH = 7.5 (40 mM HEPES), *c*_Ln_ ~ 0.2 mM, *c*_DO3A_ ~ 0.3 mM, *c*_IQCA_ ~ 0.3 mM). The self-quenching effect of NADPH ligand is also demonstrated (**d**). *λ*_em_NADPH_ = 460 nm, *λ*_em_EuL_ = 618 nm, *λ*_em_TbL_ = 545 nm, *λ*_em_SmL_ = 597 nm.

The NAD(P)H/NAD(P)^+^ redox pair plays an important role as a donor/acceptor couple in many biological processes [31,32]. The two differ by the presence of a phosphate group (NADPH/NADP^+^ vs. NADH/NAD^+^). In both cases, the reduced NAD(P)H form exhibits a broad absorption band with a maximum at about 340 nm. This can be used for excitation, with the emission band resulting in a maximum of about 460 nm. In contrast, the oxidized form does not absorb in the range of 300–400 nm and therefore does not exhibit any luminescence. The quantification of NAD(P)H is possible by luminescence measurements in the concentration range of 0–500 mg·mL^−1^ (0–1 mM), while the linear dynamic concentration range is observed for the narrower range of 0–50 mg·mL^−1^ (0–0.1 mM) [35]—see Figure 4d). In order to fit the experimental data to a suitable mathematical function for a calibration curve [35], an exponential function should be applied because of the self-quenching effect.

**Figure 5:**
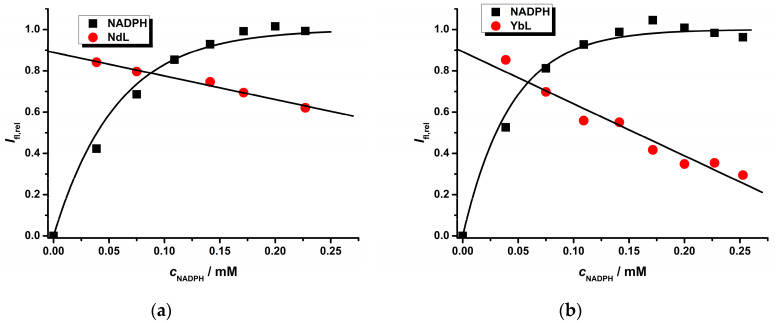
The quenching effect of NADPH on luminescence intensity of ternary Nd(III) (**a**) and Yb(III) (**b**) complexes with DO3A macrocyclic and IQCA ligands (*λ*_exc_ = 325 nm, pH = 7.5 (40 mM HEPES), *c*_Ln_ ~ 0.2 mM, *c*_DO3A_ ~ 0.35 mM, *c*_IQCA_ ~ 0.2 mM). *λ*_em_NADPH_ = 460 nm, *λ*_em_NdL_ = 880 nm, *λ*_em_YbL_ = 980 nm.

The studied Ln(III) complexes exhibit interesting physico-chemical properties due to the antenna effect (see Figure 1 and Figure 2). The IQCA antenna ligand absorbs in UV region and shows a broad excitation spectrum from 250–340 nm with several bands at 287, 296, 310 and 325 nm (Figure 1c). These wavelengths can be used for energy transfer from the ligand to Ln(III). There is a high probability that they can be influenced by the presence of NAD(P)H since both excitation bands of NAD(P)H and of ternary Ln(III) complexes significantly overlap. This quenching phenomena was studied for the chosen Ln(III) complexes (Eu, Tb, Sm, Yb, Nd)—see Figure 4 and Figure 5 and Figures S4–S8 in Supplementary Informations. The *I*_0_/*I* transformation as a function of NADPH concentration is linear (Figures S4–S8) and the slope of this dependence as *K*_Q_ is given in Table 1. Surprisingly, the highest quenching effect was observed for the Yb(III) complex and comparable with Eu(III) complex (Yb ~ Eu > Tb > Sm > Nd) while the lowest was found for the Nd(III) complex.

**Figure 6:**
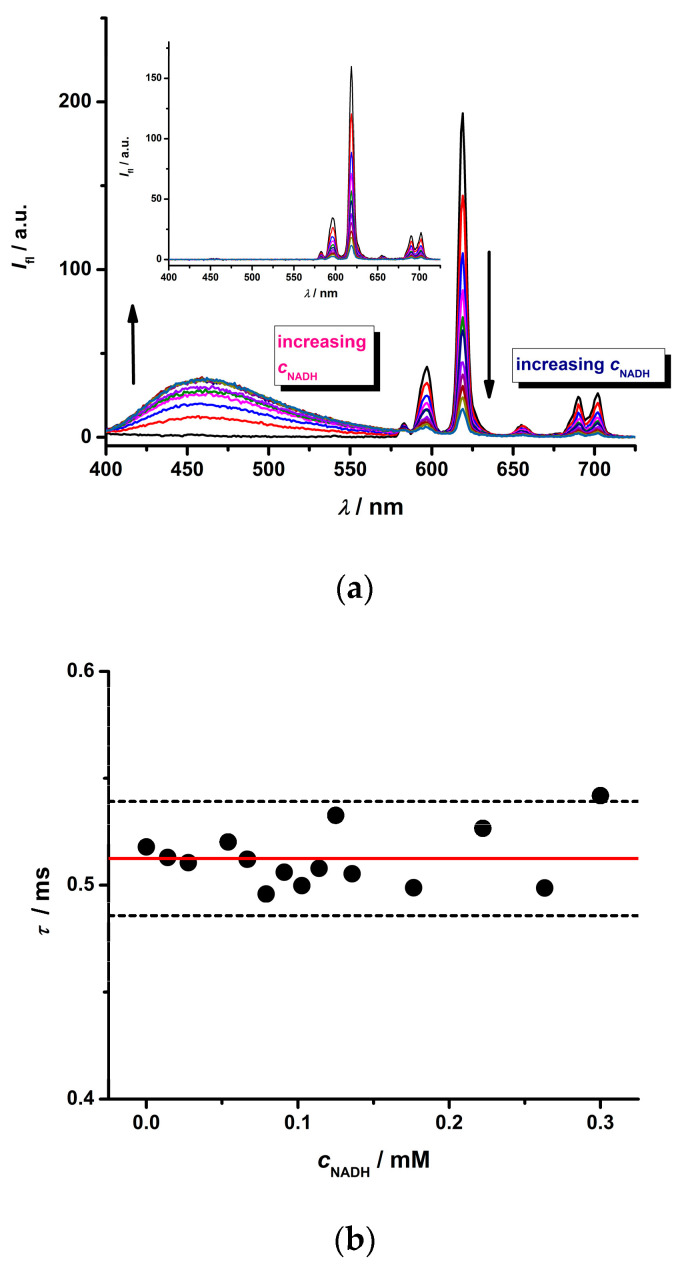
(**a**) Luminescence spectra of the Eu(III) ternary complex in the presence of NADH (the concentration in μM scale: 0, 27, 50, 66, 80,92, 99, 117, 127, 133, 158) for gating time 0 and 100 ms. (**b**) The luminescence decay of the Eu(III) ternary complex measured with the increased NADH^+^ concentration (the average value is equal to 0.512 ± 0.013 ms). The experimental conditions (*c*_EuL_, *c*_IQCA_, pH) are the same as in Figure 7.

As can be seen in Table 1, this quenching effect is not dependent on the quantum yield of the Ln(III) complexes. Further, the quenching effect is likely not caused by resonance energy transfer (RET) for the Eu(III), Tb(III), Sm(III) and Nd(III) complexes because the emission-calibration curve for NADPH shows almost the same parameters as when Ln(III) complexes are not present in solution. Additional proof is seen in the luminescence decay of the Eu(III) complex, which does not change significantly within a margin of measurement error (it was measured for the Eu(III) complex with NADH—see Figure 6b).

RET is also not probable due to the fact that direct excitation of the Ln(III) complexes in the UV range (300–405 nm) is very weak [5] and therefore, it is assumed that it is not sufficient. In the case of the Yb(III) complex, the “sensitization” of NADPH luminescence at 460 nm was also observed. This effect can be explained by a photo-redox reaction of the Yb(III) ion via the NADPH-IQCA pair. An analogous phenomenon of the sensitization of tryptophan (Trp) luminescence of the parvalbumin protein in the presence of Yb(III) ions via the {Trp^*^-Yb^3+^}-{Trp^+^-Yb^2+^}-{Trp-Yb^3+*^}-{Trp-Yb^3+^} pathways was proposed [45].

The quenching effect of the NAD(P)H ligand on the luminescence of the studied Ln(III) complexes can be explained by the shielding phenomena (see the Appendix A for mathematical proof), where the following simplified equation was derived:

*I*_fl,LnL_rel_ = ***a*** − ***b*** × *c*_NAD(P)H_ (1)

where ***a*** = ln(10) × *l* × *ε*_LnL_ × *c*_LnL_ and ***b*** = ln(10) × *l* × *ε*_NAD(P)H_. In all experiments, the relative luminescence measurements are very suitable because the differences in quantum yields of the studied Ln(III) complexes as well as the differences in experimental set-up for luminescence-spectroscopic measurements can be eliminated and the results obtained for various Ln(III) complexes can be compared.

This equation was used to fit experimental data to explain the shielding—“quenching” effect of NADPH. As can be seen in Table 1, the slope (***b***) as well as intercept (***a***) parameters are almost the same for all four Ln(III) complexes (except for Nd(III) complexes) due to the fact that the molar absorptivity of both NADPH and LnL are constant as functions of the Ln(III) complex concentration. Knowing these parameters, one can estimate the highest concentration of NADPH corresponding to a total bleaching effect on luminescence of the Ln(III) complexes, i.e., 0.32 mM (Eu), 0.39 mM (Tb), 0.45 mM (Sm), 0.78 mM (Nd), and 0.35 mM (Yb). Therefore, it can be assumed that the decrease of luminescence intensity of the Ln(III) complex can be caused by the “shielding-umbrella” effect since the light needed for excitation of the Ln(III) complex is used for NADPH’s own excitation. This general shielding effect for all Ln(III) complexes can be utilized to enlarge the dynamic concentration range of NADPH (see Figure 5 and Figure 6), but generally it is preferred to use Ln(III) complexes with higher quantum yield (mostly Eu and Tb) for luminescence measurements with higher S/N ratios.

### 2.3. Photophysical Study of the Eu(III) Complex in the Presence of an NADH/NAD^+^ Pair

To verify this shielding—“quenching” phenomenon described above, additional experiments were carried out with the Eu(III) ternary complex, which was chosen for several reasons. Firstly, due to its high Stokes shift, the emission spectrum of the Eu(III) complex does not overlap with the broad emission spectrum of NADH (see Figure 6a), as in case of the Tb(III) complex (Figure S9). Secondly, the Eu(III) ternary complex has almost the highest quantum yield, which enables the measurement of emission spectra with low error (compare Figure 2 and Figure 4). In addition, NADH was selected to elucidate the effect of the phosphate group in the NADH moiety on Ln(III) complex luminescence.

Increasing NADH concentration in a solution containing Eu(III) ternary complex leads to higher NADH luminescence intensity represented by a broader emission band in the 400–575 nm range (Figure 6a). The opposite effect was observed for the sharp peaks of the Eu(III) ternary complex detected at about 600, 620, 650 and 700 nm. The total quenching of Eu(III) complex emission was observed at an NADH concentration of about 0.3 mM; this value is of the same order of magnitude as for NADPH (0.32 mM). This is proof that the phosphate group does not play an important role in the shielding—quenching phenomenon.

**Figure 7:**
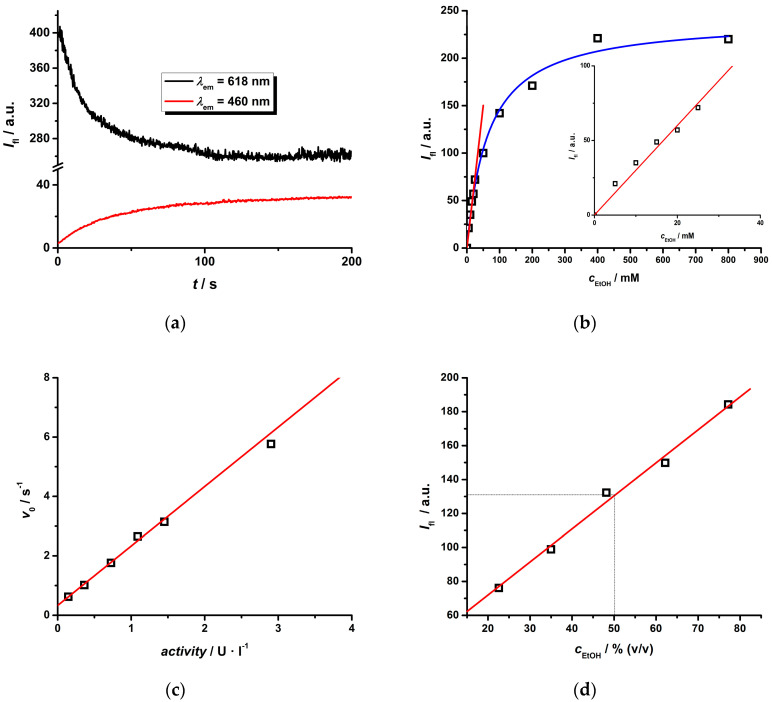
The enzymatic reaction of ethanol transformation catalyzed by the ADH enzyme (pH = 8.0 (20 mM HEPES), *c*_EuL_ = 0.1 mM, *c*_IQCA_ = 0.15 mM, *c*_NAD_ = 0.1 mM). (**a**) an example of the time trace of fluorescence intensity for the enzymatic reaction under the same experimental conditions (*c*_EtOH_ = 0.1 M). (**b**) the dependence of fluorescence intensity change of the Eu(III) complex on ethanol substrate measured at the end of enzymatic reaction (ADH_activity = 1.5 U.L^−1^). (**c**) the dependence of the initial rate of the enzymatic reaction on enzyme ADH activity (*c*_EtOH_ = 0.1 M). The initial rate was calculated from the time dependence of fluorescence intensity of Eu(III) complex in the course of the enzymatic reaction—see Figure 7a. (**d**) an example of the analysis of a real sample of apricot brandy “meruňkovice”.

Since some small contribution of NADH to the overall fluorescence signal can be expected, the time-gated emission spectra with a delay of 100 ms were recorded (inset in Figure 6a). One can observe that the emission spectra of NADH disappeared while the intensity of the luminescence spectra belonging to the Eu(III) complex slightly decreased. Numerical analysis of both experimental data given in Figure 6a enables calculation of the slope (sensitivity) of both calibration plots: 930 ± 80 mM^−1^ (gate time 0 ms) vs. 850 ± 70 mM^−1^ (gate time 100 ms). As can be noticed, the slopes do not significantly differ from a statistical point of view, and therefore the measurements can be done in both modes. The time-gated mode will likely be applied in cases when it is assumed that the emission spectra of both NADH and the Ln(III) complex significantly overlap (see Figure S9—Tb(III) and/or Dy(III)/Sm(III) complexes with low quantum yield). In the opposite case, the simultaneous dual measurements of fluorescence signals for both NADH and Eu(III) complex can be done (Figure 7a). In comparison to the slope of 480 ± 20 mM^−1^ obtained from *I* = f(*c*_NADH_) measured at 460 nm, one can see higher sensitivity of the calibration plot for NADH using the Eu(III) ternary complex.

### 2.4. Cascade Enzymatic Reaction

In order to apply the information attained above, the Eu(III) ternary complex was utilized as a probe for an enzymatic reaction. The following reaction:

CH_3_CH_2_OH + NAD^+^ → CH_3_CHO + NADH + H^+^ (R5)

was chosen as an example since it is catalyzed by the ADH enzyme and NAD^+^ is utilized as a co-factor. In the course of the enzymatically catalyzed reaction, NAD^+^ is transformed into NADH, which can be directly detected by its own absorbance/fluorescence signal and/or indirectly via luminescence of the Ln(III) complex. Because of the possibility of overlapping emission spectra for NADH and some Ln(III) complexes, which cannot be eliminated by time-gated spectra measurements leading to the decrease of the fluorescence signal, the Eu(III) ternary complex was chosen.

ADH is a suitable enzyme to determine the content of ethanol. The enzymatic biochemical analysis can be carried out at two wavelengths (460 and 618 nm) during which the time-dependence of the luminescence signal is measured. Firstly, the initial rate of the enzymatic reaction can be estimated and then used to evaluate the *v*_0_ = f (*c*_EtOH_) dependence. Secondly, the fluorescence signals of both compounds (NADH, Eu(III) complex) can be measured simultaneously (Figure 7a). This approach was employed for the construction of a calibration curve (Figure 7b), and the highest change of fluorescence signal was detected at the end of the enzymatic reaction. This plot is linear up to a 30 mM concentration, with a limit of detection of about 0.1 mM, and becomes non-linear for higher concentrations due to saturation of the active site of the AD enzyme. Thus, the non-linear equation [30]:

(2) $$∆I_{\mathrm{fl}}=∆I_{\mathrm{fl},\max}\frac{\left[S\right]}{K_{d}+\left[S\right]}$$

can be used to fit the experimental data (Figure 7b), which gives the following parameters: Δ*I*_f,max_ = 241 ± 6, *K*_d_ = (66 ± 6) mM. The slope of the linear calibration curve can be calculated as Δ*I*_f,max_*/K*_d_ = (3.6 ± 0.3) mM^−1^ which agrees with the slope (3.0 ± 0.1) mM^−1^ obtained by the fitting of the linear part (Figure 7b). The value *K*_d_ = (66 ± 6) mM is of the same order of magnitude as the Michaelis constant for the ethanol-ADH system and agrees with values given in literature [36]. In addition, it was observed that there is no inhibition effect of the Eu(III) complex on the enzymatic reaction (see Figure S10).

The initial-rate approach was also applied for ADH enzyme activity (see Figure 7c), which is an important parameter as it changes during storage and sometimes under lower temperature [46]. The linear plot can be employed for the estimation of ADH enzyme activity. The slope (sensitivity) of that calibration plot was estimated to be (2.0 ± 0.1) s^−1^.U^−1^.L with LOD ~ 0.03 U.L^−1^.

This linear concentration dependence can be applied for the analysis of real samples, such as apricot brandy “meruňkovice” (see Figure 7d). In this case, the calibration curve including the dilution factor was constructed in % (*v*/*v*) scale for practical analysis. The ethanol content of 50.3 ± 0.3% (*v*/*v*) found by the enzymatic method agrees with the value 50.1% determined by gas chromatography (GC), and no systematic error was detected. This is also caused by the fact that the ADH enzyme is more sensitive to ethanol than to methanol [36], which can be also present in brandy. Therefore, this method can be employed for the analysis of samples containing both alcohols.

## 3. Materials and Methods

The macrocyclic ligands (H_2_DO2A, H_3_DO3A) were purchased from CHEMATECH (Dijon, France). The Ln(III) chloride salts (Nd(III), Sm(III), Eu(III), Tb(III), Dy(III), Yb(III)) of analytical grade purity were purchased from Alfa-Aesar (Darmstadt, Germany). NADPH and NADH compounds and enzyme alcohol-dehydrogenase (ADH) from *Saccharomyces cerevisiae* (321 U·mg^−1^) of biochemical grade purity were purchased from Sigma-Aldrich (St. Louis, MO, USA) and their solutions were prepared fresh daily.

The emission and excitation spectra of the Ln(III) ternary complexes without and in presence of the NADPH compound were recorded on Horiba FluoroMax-4P (Eu—input/output slit 5/0.5 nm, Tb, Sm, Dy—input/output slit 5/2 nm, all integration time 100 ms) and Horiba JY Fluorolog (Yb, Nd—input/output slit 14.7/14.7 nm, integration time 300 ms) spectrofluorometers (both Kyoto, Japan) containing sensitive NIR photomultiplier detectors using air- and water-cooling. The steady-state and time-resolved luminescence studies of Eu(III) complexes in presence of the NADH ligand were carried out on the PTI spectrometer QM 300 Plus (Horiba), operating with a flash 150 W Xe-lamp (frequency 300 Hz) in the wavelength range of 200–900 nm. All emissions were corrected by the wavelength sensitivity (correction function) of the spectrometer using 395 nm filter (for the visible region) and 600 nm (for the NIR region). All measurements were performed at laboratory temperature (~298.2 K).

The HRMS spectra of aqueous solutions of [Ln(DO3A)(IQCA)] complexes (pH ~ 6.0) were recorded on a 6224 Accurate-Mass TOF LC-MS system (Agilent, Santa Clara, California, USA) in negative mode using electrospray ionization (ESI) under following conditions: nitrogen flow 5 L/min, gas temperature 325 °C, nebulizer 45 psig and capillary voltage 2.5 kV.

## 4. Conclusions

In this paper, new ternary Ln(III) complexes have been investigated from a photophysical point of view. Eu(III) and Tb(III) ternary complexes of DO3A and IQCA ligands exhibit emission spectra with several characteristic peaks and high values of quantum yield, while Sm(III) and Dy(III) complexes have lower values. On the contrary, the Nd(III) and Yb(III) complexes with the lowest quantum yield values demonstrate the highest Stokes shift—550–650 nm—of all the studied Ln(III) complexes.

The “quenching” effect of NAD(P)H on the luminescence spectra of Ln(III) complexes was also investigated and it was proposed that this phenomenon is caused by the shielding of light needed for excitation of Ln(III) complexes by NAD(P)H. Due to the common excitation wavelength for both NAD(P)H and Ln(III) complexes, it is possible to measure both fluorescence signals (NAD(P)H in region 400–750 nm, Ln(III) depends on the type of Ln(III) ion). In some cases, such as Tb(III) or Sm(III), both emission spectra are overlapping with NAD(P)H; therefore, the time-gating mode should be applied to filter both signals leading to a decrease of the Ln(III) complex signal. Therefore, there seem to be more useful applications of Ln(III) complexes emitting in the NIR region, where the Stokes shift is higher, than for Ln(III) complexes emitting in the visible region. Another advantage of using Ln(III) complexes for detection is the higher sensitivity and broader linear concentration range of NAD(P)H than in case of direct fluorescence signal measurement at 460 nm.

The benefits mentioned above can be utilized for monitoring enzymatic reactions where the NAD(P)H/NAD(P)^+^ redox pair is an essential co-factor. As an example, the enzymatic transformation of ethanol into acetaldehyde catalyzed by alcohol-dehydrogenase (ADH) was chosen and the course of this reaction was monitored by the fluorescence measurement of the indirect signal of the Eu(III) ternary complex and direct signal of NAD(P)H. It was also shown that the Eu(III) complex does not have any inhibiting effect on the enzymatically catalyzed reaction, which exhibits behavior according to the mathematical model postulated by Michaelis and Menten. The calibration plot for ethanol is linear in the 0.1–30.0 mM range. The initial-rate method also enables the estimation of ADH enzyme activity. This new method was verified by the analysis of a real sample of fruit brandy and the results agree with values obtained by GC.

Thus, the results presented in this paper show that Ln(III) complexes are suitable for indirect determination of NAD(P)H compounds and can be used for detection in many biological systems where the NAD(P)H/NAD(P)^+^ redox pair plays important role, mainly in enzymatic reactions. While the Eu(III) complex is suitable for the detection of the course and end of enzymatic reactions, the Yb(III) and Nd(III) complexes should be employed in steady-state modes at the end of the reaction because of technical difficulties of fluorescent measurements in NIR region. The utilization of these Ln(III) complexes with desired photo-physical properties opens doors for future applications in biochemical and clinical analysis.

## Figures and Tables

**Table 1:** The quenching effect of NADPH on Ln(III) complexes.

Ln(III) Complex	NADPH ^a^ *k*′/mM^−1^	LnL ^b^ *K*_Q_/mM^−1^	LnL ^c^		*I*_fl,rel_^d^
			*b*_slope_/mM^−1^	*a*_intercept_	
---	7.1 ± 0.1	---	---	---	---
Eu(III)	6.3 ± 0.2	9.5 ± 0.6	2.45 ± 0.08	0.80 ± 0.01	1.000
Tb(III)	6.0 ± 0.3	5.8 ± 0.3	2.5 ± 0.1	0.97 ± 0.01	1.042
Sm(III)	6.9 ± 0.3	4.2 ± 0.1	2.2 ± 0.1	0.97 ± 0.01	0.136
Nd(III)	7.7 ± 0.3	2.2 ± 0.3	1.14 ± 0.08	0.89 ± 0.01	0.008
Yb(III)	10.1 ± 0.4	10.2 ± 0.8	2.5 ± 0.2	0.89 ± 0.04	0.025

**^a^** Calibration curve for NADPH^+^: Eq. *I*_fl,rel_ = *I*_fl_/*I*_fl,max_ = 1–10^−*k′* × cNADPH^. **^b^** Eq. *I*_fl,LnL/_*I*_fl,LnL_NADPH_ = *K*_Q_ × *c*_NADPH_ + *Q*. **^c^** Eq. *I*_fl,rel_ = *I*_fl,LnL_NADPH_/*I*_fl,LnL_ = *a* − *b* × *c*_NADPH_. **^d^** Relative value calculated with *I*_fl,EuL_ = 4.32 × 10^5^.

## Supplementary Materials

The following are available online: the HR-MS of aqueous solutions of [Eu(DO3A)(IQCA)]^−^, [Tb(DO3A)(IQCA)]^−^ and [Yb(DO3A)(IQCA)]^−^ complexes (Figures S1–S3), the quenching effect of NADPH on Ln(III) complexes (*I*_0_/*I* = f (c_NADPH)—Figures S4–S8), the effect of gating time on luminescence spectra of [Tb(DO3A)(IQCA)]^−^ complex in presence of NADPH (Figure S9), the effect of presence of Eu(III) ternary complex on the course of enzymatic reaction (Figure S10).

## Appendix A

### The Shielding Effect of NAD(P)H on Luminescence of Ln(III) Ternary Complexes

One can start to derive some basic equations using Bouger-Lambert-Beer’s law for absorption and quantum yield for luminescence emission.

The luminescence intensity of NAD(P)H can be described by the general equation:

*I*_fl,NAD(P)H_ = *k* × *φ*_NAD(P)H_ × *I*_abs,NAD(P)H_ = *k* × *φ*_NAD(P)H_ × *I*_0_ × (1 − 10^− ln(10) ×^*^ε^*^NAD(P)H ×^*^l^*^×^*^c^*^NAD(P)H^) (A1)

where some parameters are related to the experimental setup of the luminescence instrumentation (*k*—proportionality constant, mostly depends on detector (e.g., efficiency, position, distance cuvette-detector, etc.; *l*—path-length of cuvette, where is placed NAD(P)H solution)), while other parameters are related to the NAD(P)H solution (*φ*—quantum yield, *ε*—molar absorptivity, *c*—molar concentration). As can be noticed, the luminescence intensity of NAD(P)H is approaching to maximal value at higher concentration:

*I*_fl_max,NAD(P)H_ → *k* × *φ*_NAD(P)H_ × *I*_0_ (A2)

Thus, the normalized intensity, which is independent of the experimental set-up of spectro-fluorimetric instrumentation, can be obtained as:

*I*_fl,NAD(P)H_rel_ = 1 − 10^−ln(10) ×^*^ε^*^NAD(P)H ×^*^l^*^×^*^c^*^NAD(P)H^*=* 1 − 10^−*k*′ ×^*^c^*^NAD(P)H^ (A3)

This mathematical function was utilized to fit experimental normalized luminescence data as a function of NAD(P)H concentration—see Table 1. For diluted solutions, Equation (A1) can be simplified to:

*I*_fl,NAD(P)H_ = *k* × *φ*_NAD(P)H_ × *I*_0_ × (1 − 10^− ln(10) ×^*^ε^*^NAD(P)H ×^*^l^*^×^*^c^*^NAD(P)H^)
≈ ln(10) × *k* × *φ*_NAD(P)H_ × *I*_0_ × *ε*_NAD(P)H_ × *l* × *c*_NAD(P)H_*≈ k′*_NAD(P)H_*× c*_NAD(P)H_ (A4)

where *k*′ is the proportionality constant, which includes the above described parameters (*k*, *φ*_NAD(P)H_, *I*_0_, *ε*_NAD(P)H_, *l*).

Analogously to Equation (A1), one can assume that the luminescence intensity of Ln(III) complexes can be calculated as:

*I*_fl,LnL_ = *k* × *φ*_LnL_ × *I*_abs,LnL_ = *k* × *φ*_LnL_ × *I*_0_ × (1 − 10^−ln(10) × *ε* LnL × *l* × *c*LnL^)
≈ ln(10) × *k* × *φ*_LnL_ × *I*_0_ × *ε*_LnL_ × *l* × *c*_LnL_ ≈ *k*″ × *ε*_LnL_ × *c*_LnL_ (A5)

In presence of NAD(P)H, the previous equation describing the luminescence of Ln(III) complex can be modified as follows:

*I*_fl,LnL_ = *k* × *φ*_LnL_ × (*I*_abs, LnL −_*I*_abs, NAD(P)H_) = *k* × *φ*_LnL_ × *I*_0_ × (10^−ln(10) ×^*^ε^*^NAD(P)H ×^*^l^*^×^*^c^*^NAD(P)H^ − 10^−ln(10) ×^*^ε^*^LnL ×^*^l^*^×^*^c^*^LnL^) (A6)

and for diluted solutions after normalization with respect to maximal value as:

*I*_fl,LnL_rel_ = (10^− ln(10) ×^*^ε_^*^NAD(P)H ×^*^l^*^×^*^c_^*^NAD(P)H^ − 10^−ln(10) ×^*^ε^*^LnL ×^*^l^*^×^*^c^*^LnL^) ≈
≈ ln(10) × *l* × (*ε*_LnL_ × *c*_LnL_ − *ε*_NAD(P)H_ × *c*_NAD(P)H_) (A7)

Assuming that the concentration of the Ln(III) complex is constant during the experiment, the Equation (A7) can be simplified to the following equation:

*I*_fl,LnL_rel_ = ***a*** − ***b*** × *c*_NAD(P)H_ (A8)

where ***a*** = ln(10) × *l* × *ε*_LnL_ × *c*_LnL_ and ***b*** = ln(10) × *l* × *ε*_NAD(P)H_. In all experiments, the relative luminescence measurements are very suitable because the differences in the experimental set-up of the luminescence instrumentation (e.g., intensity of excitation source, input/output slit widths, sensitivity of detector, integration time, etc.) can be eliminated and the results obtained for various Ln(III) complexes can be compared.
