# Supplementary material for: Luminescent Sensor Based on Ln(III) Ternary Complexes for NAD(P)H Detection
Source: Molecules. 2020 Sep 11;25(18):4164. doi: 10.3390/molecules25184164 (PMC7571129; doi:10.3390/molecules25184164)
Supplement: Supplementary file 1 [file molecules-25-04164-s001.pdf]

Supporting Informations for

Luminescent sensor based on Ln(III) ternary complex for NAD(P)H detection

Filip Smrčka and Přemysl Lubal*

Department of Chemistry, Faculty of Science, Masaryk University, Kamenice 5, 625 00 Brno, Czech Republic
* Correspondence: lubal@chemi.muni.cz

Content

The high-resolution mass spectra (HR-MS) of chosen Ln(III) ternary complexes

Fig. S1. Ternary Eu(III) complex…………………………………………………………………**2**

Fig. S2. Ternary Tb(III) complex…………………………………………………………………**3**

Fig. S3. Ternary Yb(III) complex…………………………………………………………………**4**

The quenching effect of NADPH compound:

Fig. S4. Ternary Eu(III) complex…………………………………………………………………**5**

Fig. S5. Ternary Tb(III) complex…………………………………………………………………**5**

Fig. S6. Ternary Sm(III) complex…………………………………………………………………**6**

Fig. S7. Ternary Nd(III) complex…………………………………………………………………**6**

Fig. S8. Ternary Yb(III) complex…………………………………………………………………**7**

Fig. S9. The effect of gating time on luminescence spectra of mixture
NADPH an Tb(III) ternary complex……………………………………………………………....**7**

Fig. S10. The effect of Eu(III) complex on enzymatic reaction of ethanol transformation
catalyzed by ADH………………………………………………………………………………....**8**

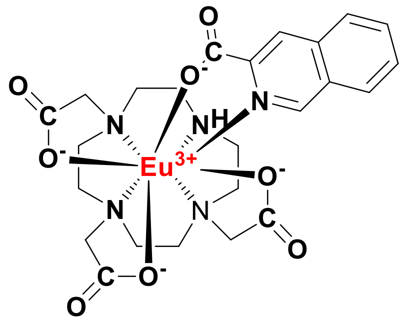

expected mass: [Eu(DO3A)(IQCA)]$^-$ = **668.1236**

observed mass: [Eu(DO3A)(IQCA)]$^-$ = **668.1231**

mass accuracy = 0.7 ppm

$C_{24}H_{29}O_8N_5Eu$ exact mass: 668.1230

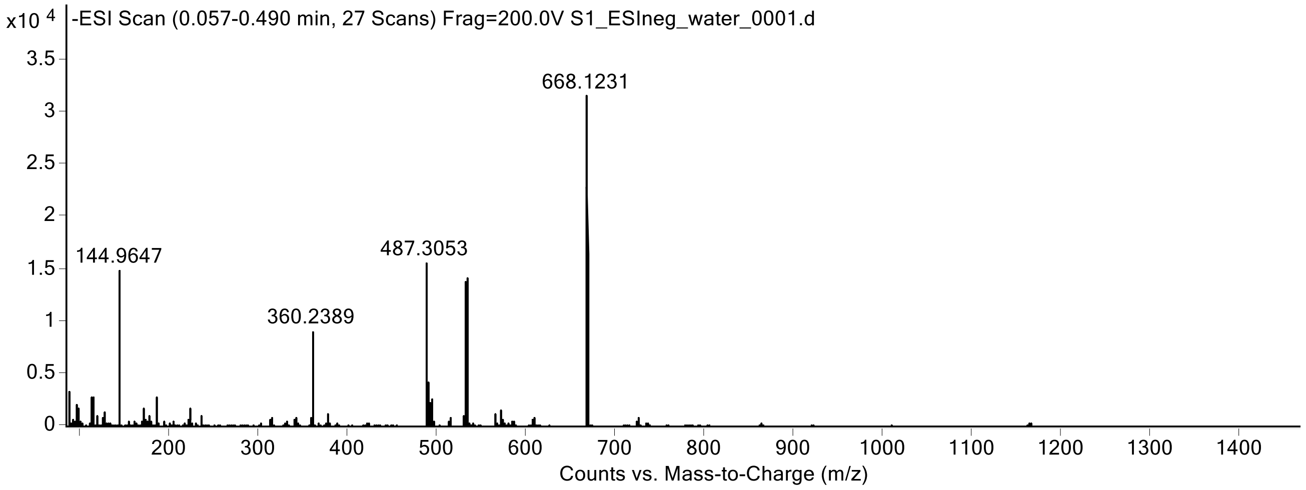

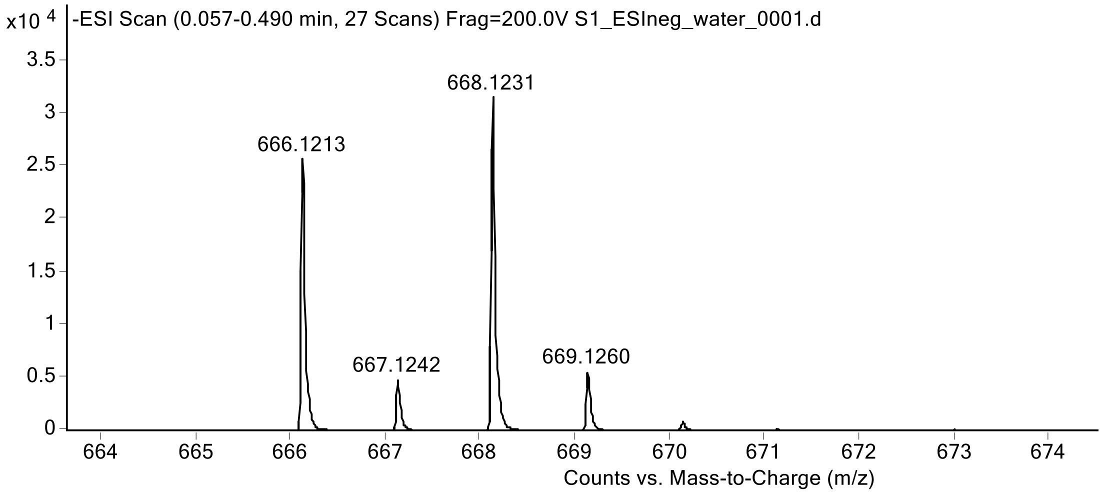

Fig. S1. The HR-MS for ternary [Eu(DO3A)(IQCA)]$^-$ complex

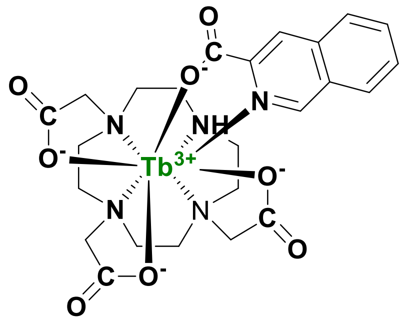

expected mass: [Tb(DO3A)(IQCA)]$^-$ = 674.1275

observed mass: [Tb(DO3A)(IQCA)]$^-$ = 674.1279

mass accuracy = - 0.6 ppm

$C_{24}H_{29}O_8N_5Tb$ exact mass: 674.1270

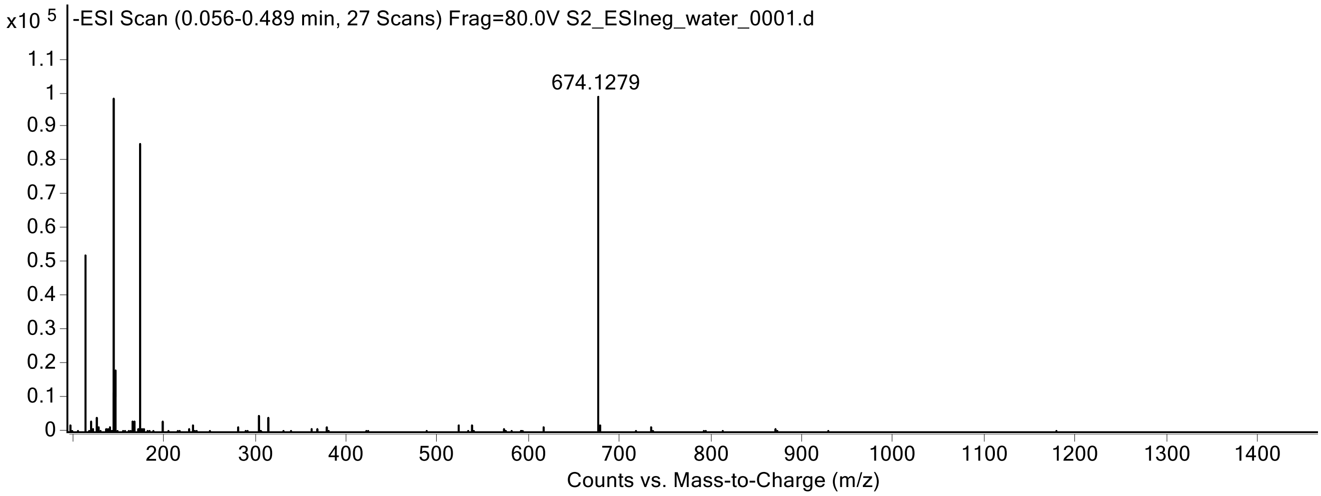

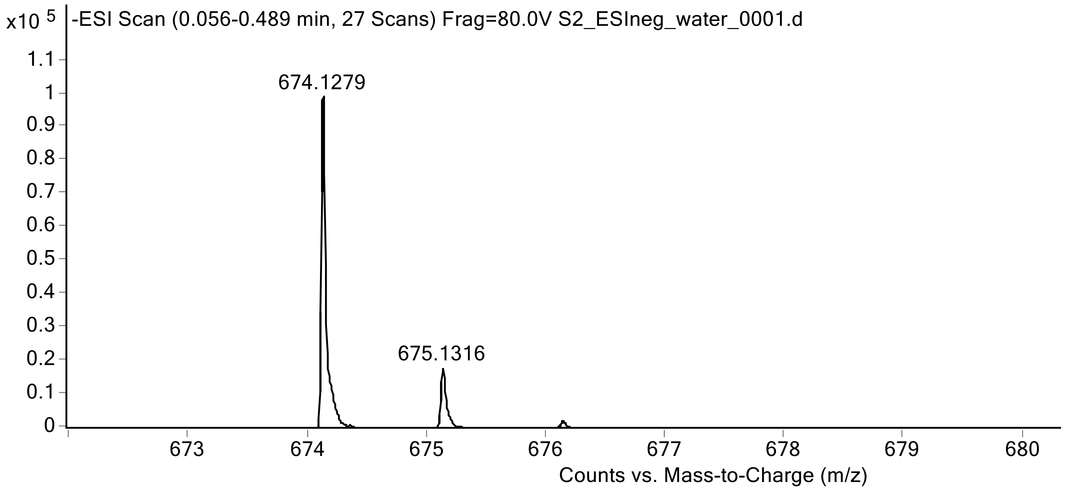

Fig. S2. The HR-MS for ternary [Tb(DO3A)(IQCA)]$^-$ complex

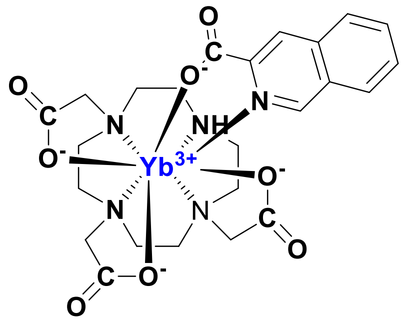

expected mass: [Yb(DO3A)(IQCA)]$^-$ = <u>689.1414</u>

observed mass: [Yb(DO3A)(IQCA)]$^-$ = <u>689.1417</u>

mass accuracy = - 0.4 ppm

$C_{24}H_{29}O_8N_5Yb$ **exact mass: 689.1405**

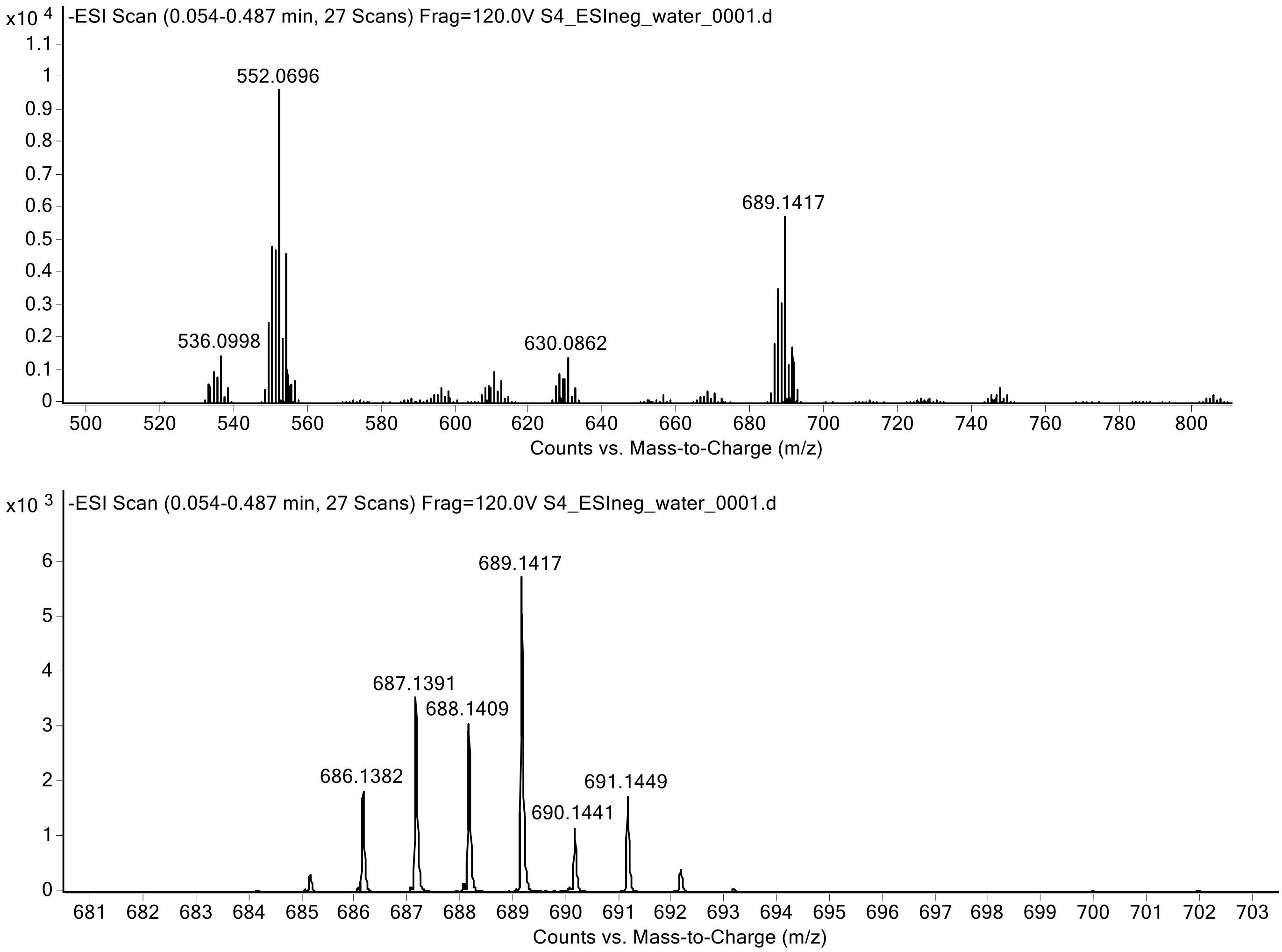

Fig. S3. The HR-MS for ternary [Yb(DO3A)(IQCA)]$^-$ complex

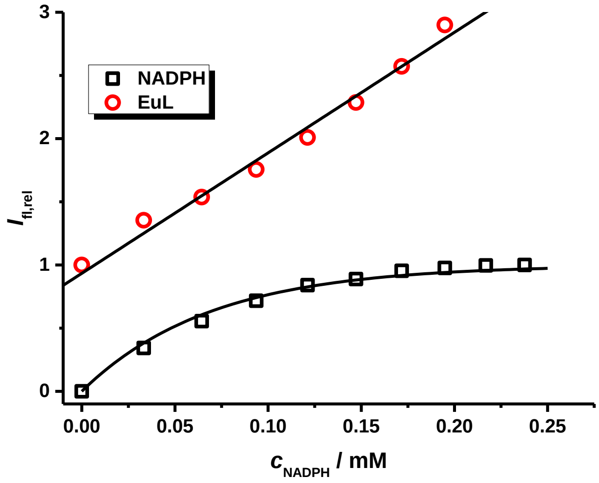

Fig. S4. The quenching effect of NADPH on luminescence intensity of ternary Eu(III) complex with DO3A macrocyclic and IQCA ligands (λ_{exc} = 325 nm, pH = 7.5 (40 mM HEPES), c_{Ln} ~ 0.2 mM, c_{DO3A} ~ 0.3 mM, c_{IQCA} ~ 0.3 mM). λ_{em_NADPH} = 460 nm, λ_{em_EuL} = 618 nm

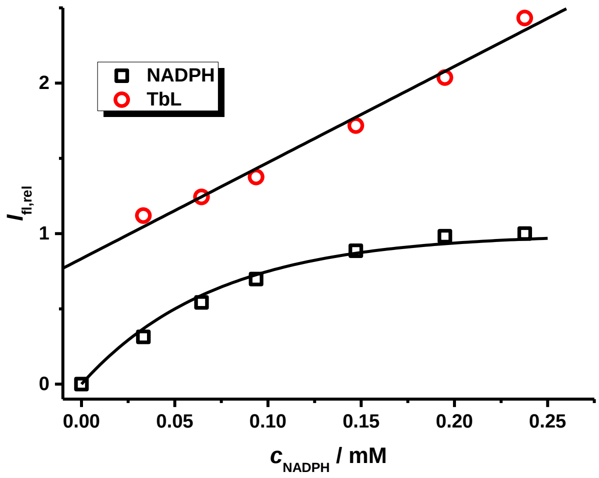

Fig. S5. The quenching effect of NADPH on luminescence intensity of ternary Tb(III) complex with DO3A macrocyclic and IQCA ligands (λ_{exc} = 325 nm, pH = 7.5 (40 mM HEPES), c_{Ln} ~ 0.2 mM, c_{DO3A} ~ 0.3 mM, c_{IQCA} ~ 0.3 mM). λ_{em_NADPH} = 460 nm, λ_{em_TbL} = 545 nm.

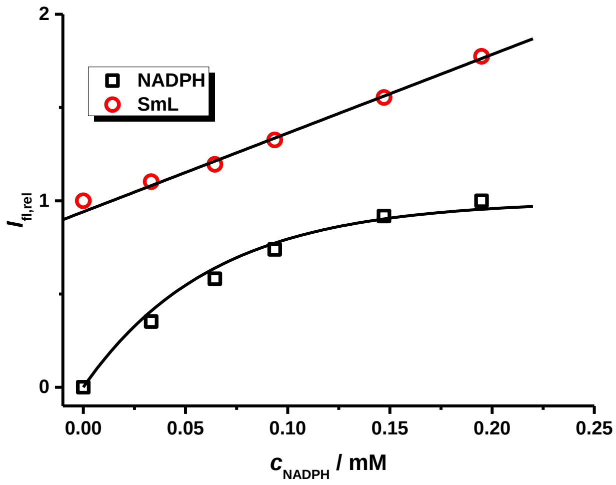

Fig. S6. The quenching effect of NADPH on luminescence intensity of ternary Sm(III) complex with DO3A macrocyclic and IQCA ligands (λ_{exc} = 325 nm, pH = 7.5 (40 mM HEPES), c_{Ln} ~ 0.2 mM, c_{DO3A} ~ 0.3 mM, c_{IQCA} ~ 0.3 mM). λ_{em_NADPH} = 460 nm, λ_{em_SmL} = 597 nm

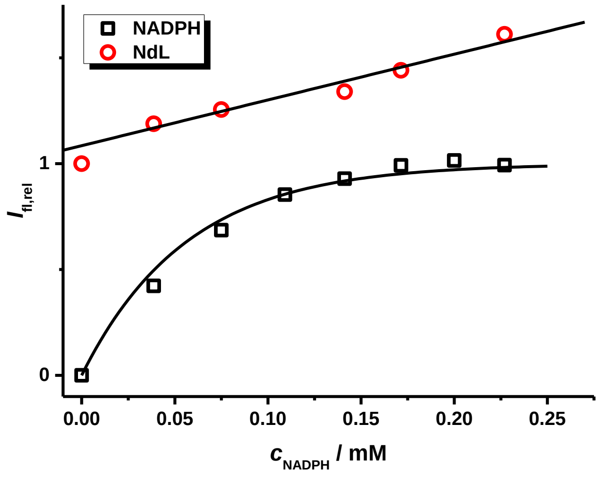

Fig. S7. The quenching effect of NADPH on luminescence intensity of ternary Nd(III) complex with DO3A macrocyclic and IQCA ligands (λ_{exc} = 325 nm, pH = 7.5 (40 mM HEPES), c_{Ln} ~ 0.2 mM, c_{DO3A} ~ 0.35 mM, c_{IQCA} ~ 0.2 mM). λ_{em_NADPH} = 460 nm, λ_{em_NdL} = 880 nm.

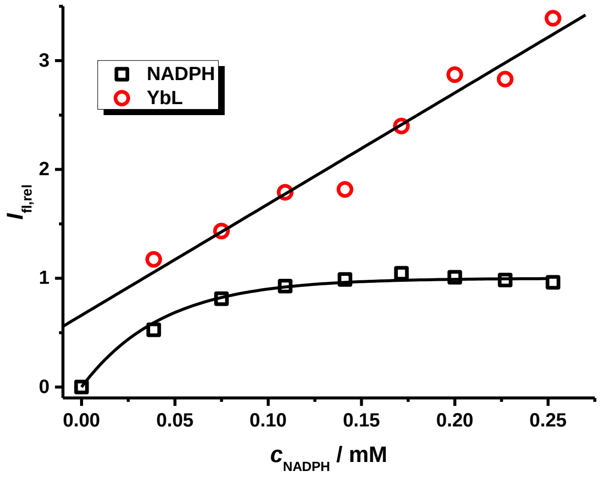

Fig. S8. The quenching effect of NADPH on luminescence intensity of ternary Yb(III) complex with DO3A macrocyclic and IQCA ligands (λ_{exc} = 325 nm, pH = 7.5 (40 mM HEPES), c_{Ln} ~ 0.2 mM, c_{DO3A} ~ 0.35 mM, c_{IQCA} ~ 0.2 mM). λ_{em_NADPH} = 460 nm, λ_{em_YbL} = 980 nm

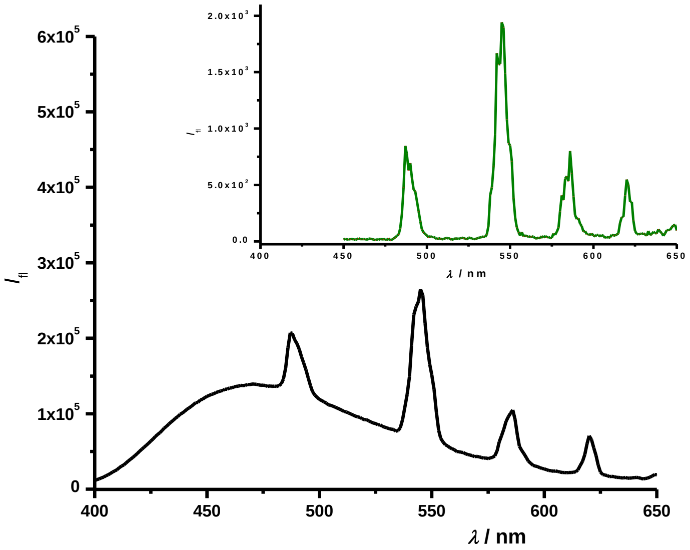

Fig. S9. The emission spectra of ternary Tb(III) complex with DO3A macrocyclic and IQCA ligands in presence of NADPH compound (λ_{exc} = 325 nm, pH = 7.5 (40 mM HEPES), c_{Tb} ~ 0.2 mM, c_{DO3A} ~ 0.35 mM, c_{IQCA} ~ 0.2 mM). The corrected spectra have not been gated (lower picture) while upper in inset were gated by Δt = 50 ms.

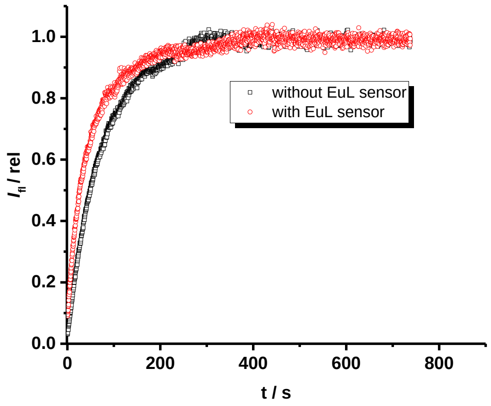

Fig. S10 The example of time trace of fluorescence intensity for enzymatic reaction under the same experimental conditions (pH = 8.0, c_{EtOH} = 0.1 M, c_{EuLZ} = 0.1 mM, c_{NAD} = 0.1 mM, λ_{exc} = 325 nm, λ_{em} = 460 nm
